# A tool for translating polygenic scores onto the absolute scale using summary statistics

**DOI:** 10.1038/s41431-021-01028-z

**Published:** 2022-01-04

**Authors:** Oliver Pain, Alexandra C. Gillett, Jehannine C. Austin, Lasse Folkersen, Cathryn M. Lewis

**Affiliations:** 1 Social, Genetic and Developmental Psychiatry Centre, Institute of Psychiatry, Psychology and Neuroscience, King’s College London, London, UK; 2 NIHR Maudsley Biomedical Research Centre, South London and Maudsley NHS Trust, London, SE5 8AF UK; 3 Department of Psychiatry and Medical Genetics, University of British Columbia, Vancouver, BC Canada; 4 Danish National Genome Center, Copenhagen, Denmark; 5 Department of Medical and Molecular Genetics, Faculty of Life Sciences and Medicine, King’s College London, London, UK

**Keywords:** Personalized medicine, Medical genomics

## Abstract

There is growing interest in the clinical application of polygenic scores as their predictive utility increases for a range of health-related phenotypes. However, providing polygenic score predictions on the absolute scale is an important step for their safe interpretation. We have developed a method to convert polygenic scores to the absolute scale for binary and normally distributed phenotypes. This method uses summary statistics, requiring only the area-under-the-ROC curve (AUC) or variance explained (*R*^2^) by the polygenic score, and the prevalence of binary phenotypes, or mean and standard deviation of normally distributed phenotypes. Polygenic scores are converted using normal distribution theory. We also evaluate methods for estimating polygenic score AUC/*R*^2^ from genome-wide association study (GWAS) summary statistics alone. We validate the absolute risk conversion and AUC/*R*^2^ estimation using data for eight binary and three continuous phenotypes in the UK Biobank sample. When the AUC/*R*^2^ of the polygenic score is known, the observed and estimated absolute values were highly concordant. Estimates of AUC/*R*^2^ from the lassosum pseudovalidation method were most similar to the observed AUC/*R*^2^ values, though estimated values deviated substantially from the observed for autoimmune disorders. This study enables accurate interpretation of polygenic scores using only summary statistics, providing a useful tool for educational and clinical purposes. Furthermore, we have created interactive webtools implementing the conversion to the absolute (https://opain.github.io/GenoPred/PRS_to_Abs_tool.html). Several further barriers must be addressed before clinical implementation of polygenic scores, such as ensuring target individuals are well represented by the GWAS sample.

## Introduction

A substantial proportion of individual differences in health and disease are explained by genetic variation [1]. Genome-wide association studies (GWAS) have successfully identified thousands of genetic loci associated with a broad range of phenotypes, from anthropometric traits such as height [2], to psychiatric disorders such as major depression [3]. One application of GWAS results is the estimation of genetic risk/propensity for a given phenotype using polygenic scores. Polygenic scores are calculated as the GWAS effect size-weighted sum of alleles carried by an individual [4]. A range of methods exist for processing GWAS summary statistics prior to polygenic scoring to account for the linkage disequilibrium (LD) between variants and improve the predictive utility of polygenic scores [5].

Polygenic scores capture only part of the genetic liability to a disease or trait, but the proportion explained increases with larger GWAS sample sizes and with improvements in polygenic scoring methodology. For example, individuals in the top 8% of cardiovascular disease (CAD) polygenic scores have a three-fold increased risk of developing CAD compared to the general population [6]. The implementation of polygenic scores within a clinical setting are being increasingly discussed and investigated [7, 8], but several barriers exist before these scores can become an established part of healthcare, including the low variance of risk explained and issues with interpretation.

For correct interpretation of a polygenic score, the score must first be standardised according to an ancestry-matched reference, so that the score is transformed to units of standard deviations (SD) from the mean. This standardised score, referred to as a polygenic *Z*-score, can then be used to calculate the relative risk of disease for an individual, based on their polygenic score. Whilst relative risk estimates are of interest, they are challenging to interpret as they do not consider the predictive utility of the polygenic score or the prevalence of the outcome in the general population. It is well established that differences in risk are most accurately perceived by non-experts when presenting risk on the absolute scale, i.e., the probability an individual will develop the outcome [9, 10]. The absolute risk conferred by a given relative risk is determined by the predictive utility of the polygenic score and the population prevalence of the phenotype. For example, an individual’s polygenic *Z*-score for schizophrenia may be 1.96, indicating their polygenic score is higher than 97.5% of an ancestry-matched population. However, the absolute risk, or probability of the individual developing schizophrenia, is unknown, as we must account for the predictive utility of the polygenic score and the population prevalence of schizophrenia.

When individual-level data are available for the polygenic score and the outcome of interest, it is possible to calculate the absolute risk conferred by a given polygenic score by measuring the proportion of cases within polygenic scores quantiles. This approach is often used in research to put variance explained estimates into perspective [6], and it is also implemented by 23andMe to help interpretation of results by their customers [11]. However, individual-level data for the phenotype of interest within a representative sample is often unavailable. Therefore, a summary statistic-based approach, requiring only information describing the predictive utility of the polygenic score and the prevalence of the outcome, would greatly improve the availability of interpretable results from polygenic scores.

Within a homogenous population, polygenic scores are normally distributed due to the central limit theorem, and therefore normal distribution theory can be used to define polygenic scores based on the distribution of the phenotype and predictive utility of the polygenic score. Using this approach, our study develops a summary statistic-based tool for converting polygenic scores into absolute estimates for both binary and normally distributed phenotypes. For binary phenotypes, we calculate absolute risk based on the predictive utility of the polygenic score and the population prevalence of the phenotype. For normally distributed phenotypes, we calculate predictions in absolute terms based on the predictive utility of the polygenic scores and the population mean and SD of the phenotype. In addition, we compare several approaches that estimate the predictive utility of polygenic scores using GWAS summary statistics alone, as this value is often unknown.

Another important factor reported to help accurately interpret differences in risk is the use of simple visual aids [9]. Therefore, this study also develops interactive webtools for converting an individual’s polygenic scores to the absolute scale with corresponding graphics.

## Materials and methods

### UK Biobank (UKB)

UKB is a prospective cohort study that recruited >500,000 individuals aged between 40 and 69 years across the United Kingdom [12]. The UKB received ethical approval from the North West—Haydock Research Ethics Committee (reference 16/NW/0274).

#### Phenotype data

Eleven UKB phenotypes with well-powered and independent GWAS summary statistics were selected to represent a range of genetic architectures (heritability/polygenicity). Eight phenotypes were binary: depression, type 2 diabetes, coronary artery disease (CAD), inflammatory bowel disease (IBD), rheumatoid arthritis (RheuArth), multiple sclerosis (MultiScler), breast cancer, and prostate cancer. Three phenotypes were continuous: intelligence, height, and body mass index (BMI). Further information regarding phenotype definitions can be found in the Supplementary Material.

Analysis was performed on a subset of ~50,000 UKB participants for each outcome to the reduce computational burden of subsequent analyses whilst maintaining sufficient statistical power. For each continuous trait (Intelligence, Height, BMI), a random sample ~50,000 individuals were selected. For disease traits, all available cases were included, except for high prevalence disease traits, Depression and CAD, where a random sample of 25,000 cases was selected. Controls were randomly selected to obtain a total sample size of 50,000. This sampling procedure was performed once for each phenotype. Sample sizes for each phenotype after genotype data quality control are shown in Table 1.

**Table 1:** Comparison between observed and estimated values on the absolute scale across polygenic score quantiles for binary and normally distributed phenotypes.

Phenotype	Mean Abs. Diff.	Mean Abs. Diff. of SD	*N*	*N*cas	*N*con	Skewness
Binary						
Depression	1.5%	NA	49,999	24,999	25,000	NA
T2D	2.4%	NA	49,999	14,888	35,111	NA
CAD	1.5%	NA	49,999	25,000	24,999	NA
IBD	6.8%	NA	49,999	3461	46,538	NA
MultiScler	12.6%	NA	49,999	1137	48,862	NA
RheuArth	6.8%	NA	49,999	3408	46,591	NA
Breast_Cancer	4.6%	NA	49,999	8512	41,487	NA
Prostate_Cancer	8.7%	NA	50,000	2927	47,073	NA
Continuous						
Intelligence	0.3%	1.3%	50,000	NA	NA	0.144
Height	0.1%	1.5%	49,999	NA	NA	0.117
BMI	0.2%	6%	49,999	NA	NA	0.592

Estimated values are based on the observed AUC/*R*^2^ of the polygenic score, the prevalence of binary phenotypes, and mean and standard deviation (SD) of continuous phenotypes in UKB.

*Mean Abs. Diff.* mean absolute difference between expected and observed case probability (binary) or trait mean (continuous), *Mean Abs. Diff. of SD* mean absolute difference between expected and observed trait standard deviation, *N* sample size, *Ncas* number of cases, *Ncon* number of controls.

#### Genetic data

UKB released imputed dosage data for 488,377 individuals and ~96 million variants, generated using IMPUTE4 software [12] with the Haplotype Reference Consortium reference panel [13] and the UK10K Consortium reference panel [14]. This study retained individuals that were of European ancestry based on 4-means clustering on the first 2 principal components provided by the UKB, had congruent genetic and self-reported sex, passed UKB test for excessive heterozygosity or missingness (variable ID = “het.missing.outliers”), and removed related individuals (>3rd degree relative, KING threshold >0.044) using relatedness kinship (KING) estimates provided by the UKB [12]. The imputed dosages were converted from BGEN v1.2 format to PLINK1 binaries (.bed/.bim/.fam) hard-call format using QCTOOL v2 (see URLs) without specifying a hard-call threshold (i.e. threshold = 0) to retain the best guess for all genotypes.

### Polygenic scoring

Polygenic scores were derived within a reference-standardised framework, whereby polygenic scores are derived using a common set of genetic variants, LD estimates, and allele frequency estimates [5]. This approach is well suited for the clinical setting and is also good practice for research purposes.

#### SNP-level QC

HapMap 3 variants from the LD-score regression website (see Web Resources) were extracted from UKB, inserting any HapMap 3 variants that were not available in UKB as missing genotypes (as required for reference MAF imputation by the PLINK allelic scoring function) [15]. No other SNP-level QC was performed.

#### Individual-level QC

Individuals of European ancestry were retained for polygenic score analysis. In addition to the 4-means clustering approach described above, we determined whether UKB participants were of European ancestry using 1000 Genomes Phase 3 projected principal components of population structure, retaining only those within three SD from the mean for the top 100 principal components. This process will also remove individuals who are outliers due to technical genotyping or imputation errors.

#### GWAS summary statistics

Publicly available GWAS summary statistics were identified for the phenotypes as defined above, or similar phenotypes (descriptive statistics in Table S1) [2, 16–25]. We excluded GWAS with documented sample overlap with UKB. Quality control of GWAS summary statistics is described in the Supplementary Material.

#### Reference genotype datasets

Genotype-based scoring in UKB was reference-standardised using the European subset of 1000 Genomes Phase 3 (*N* = 503) as the reference. This means the GWAS summary statistics were processed for polygenic scoring using only reference genotype data to estimate LD and allele frequencies, and the resulting polygenic scores in UKB were scaled and centred according to the mean and SD of the score in the reference. The reference-standardised approach has been described in further detail previously [5].

#### Polygenic scoring methodology

Polygenic scoring was carried out using DBSLMM [26], which models LD between genetic variants and applies shrinkage parameters to avoid overfitting. DBSLMM is a computationally scalable method that performs similarly to other leading polygenic scoring methods [5]. For comparison with the DBSLMM polygenic score results, a threshold and clump (pT + clump) approach was also used.

pT + clump was performed using an *R*^2^ threshold of 0.1 and window of 250 kb. Within the MHC region (28–34 Mb on chromosome 6), the pT + clump method retains only the single most significant variant due to long range and complex LD in this region. Ten *p* value thresholds were used to select variants: 1 × 10^−8^, 1 × 10^−6^, 1 × 10^−4^, 1 × 10^−2^, 0.1, 0.2, 0.3, 0.4, 0.5 and 1. The pT + clump approach was implemented using PLINK v1.9 [15].

After preparation of GWAS summary statistics, polygenic scores were calculated using PLINK with reference MAF imputation of missing data. All scores were standardised (scaled and centred) based on the mean and SD of polygenic scores in the reference sample (European subset of 1000 Genomes Phase 3).

### Converting from relative to absolute scale

Converting to the absolute scale here requires updating the distribution parameters for a phenotype, given that we observe a polygenic score within a specified range (determined by quantiles). We develop methodology to achieve this for both binary and normally distributed phenotypes, using only the distribution of the phenotype and the predictive utility of the polygenic score.

Broadly, our approach defines the population distribution for the polygenic score using a measure of its predictive utility within normal distribution theory. The polygenic score quantiles are then estimated and, using these and the phenotype and polygenic score distributions, we derived the required, updated distribution parameters for the phenotype using conditional probability rules.

For binary phenotypes, such as major depression and MultiScler, polygenic scores can be modelled as a mixture of two normal distributions, using the population prevalence of the phenotype, and the predictive utility of the polygenic scores, often indicated by the area-under-the-ROC-curve (AUC). Once this distribution has been defined, the quantiles of the polygenic scores, and the proportion of cases within each quantile is estimated. Quantile estimation for the mixture distribution requires using a root-finding algorithm; here we use the “uniroot” function in the “base” R package [27]. A full derivation of the formulae for conversion to the absolute scale is available in the Supplementary Material.

For normally distributed continuous phenotypes, such as height and IQ, polygenic scores are defined as part of a bivariate normal distribution with the phenotype, using the mean and SD of the phenotype in the general population, and the predictive utility of the polygenic scores, often indicated as the variance explained (*R*^2^). Once this distribution has been defined, the quantiles of the polygenic scores, and the phenotype mean and SD within each quantile is estimated. The mean and SD of the phenotype within each polygenic score quantile are estimated using the “mtmvnorm” function in the “tmvtnorm” R package [28]. A full derivation of the formulae for the conversion to the absolute scale is available in the Supplementary Material.

Both conversions use some assumption of normality when defining the distribution of the polygenic score, which is underpinned by the central limit theorem. We note that polygenic scores derived using the pT + clump approach will often include only a small number of genetic variants when using a stringent *p* value threshold and may therefore not fit a normal distribution. To determine whether the conversions are biased in this scenario, we compared absolute estimates to those observed when using pT + clump polygenic scores based on the most stringent p-values threshold available, whilst retaining at least five genetic variants.

### Estimating predictive utility of polygenic scores

The *pseudovalidate* function of the “lassosum” R package [29] estimates the correlation (*R*) between the polygenic score and GWAS phenotype to identify the optimal lassosum hyper parameters (s and lambda). We have previously shown lassosum has a similar predictive utility to the DBSLMM polygenic score [5]. For continuous phenotypes, the variance explained by the polygenic scores is *R*^*2*^. For binary phenotypes, the AUC is obtained from the correlation via calculation of the Cohen’s *d*, accounting for the GWAS sampling ratio [30, 31]. The conversion of *R* to Cohen’s *d* is described in the Supplementary Material.

In the Supplementary Material, we explore two other approaches for estimating the predictive utility of polygenic scores, including AVENGEME [32] and G-WIZ [33].

### Validation procedure

#### Conversion to absolute scale

Conversion to the absolute scale for binary and continuous phenotypes was validated in UKB. For binary phenotypes, the conversion was validated by comparing the observed number of affected individuals within each polygenic score quantile to the estimated values. For continuous phenotypes, the conversion was validated by comparing the observed phenotype mean and SD within each polygenic score quantile to estimated values. When validating the conversion to the absolute scale, the observed AUC/*R*^2^ of the polygenic score, and observed sampling ratio or phenotype mean and SD were used to estimate the polygenic score distribution. We also compared observed and estimated values for Height stratified by sex.

#### Estimation of polygenic score AUC/*R*^2^

To validate the AUC and *R*^*2*^ estimates derived from lassosum, we compared the estimated values to those observed in UKB. We also used the estimated AUC/*R*^2^ values when converting polygenic score into absolute terms, to determine the extent to which differences between observed and estimated AUC/*R*^2^ values influenced the results.

### Development of interactive visualisation tool

We developed an interactive webtool for converting and visualising standardised polygenic scores to the absolute scale for binary and normally distributed phenotypes. The webtools were developed using the “shiny” package in R, and are hosted on the shinyapps.io website (see URLs). For the shiny app implementation of the absolute scale conversion, the polygenic score distribution is split into 1000 quantiles to increase the precision of the results.

Databases reporting the predictive utility of polygenic scores, such as the Polygenic Score Catalog [34] (see URLs), store a range of effect size metrics for polygenic scores of binary outcomes, including AUC, *R*^*2*^ on the observed scale, *R*^*2*^ on the liability scale, odds ratio per standard deviation, and Cohen’s *d*. To enhance the utility of the interactive webtool for binary outcomes, we allow the predictive utility of the polygenic scores to be specified using any of these metrics. Details for the conversion between odds ratio per standard deviation and Cohen’s *d* are provided in the Supplementary Material. Other conversions have been previously documented: AUC [31], *R*^*2*^ on the observed scale [30], *R*^*2*^ on the liability scale [35].

## Results

### Validating conversion to absolute terms

**Fig. 1:**
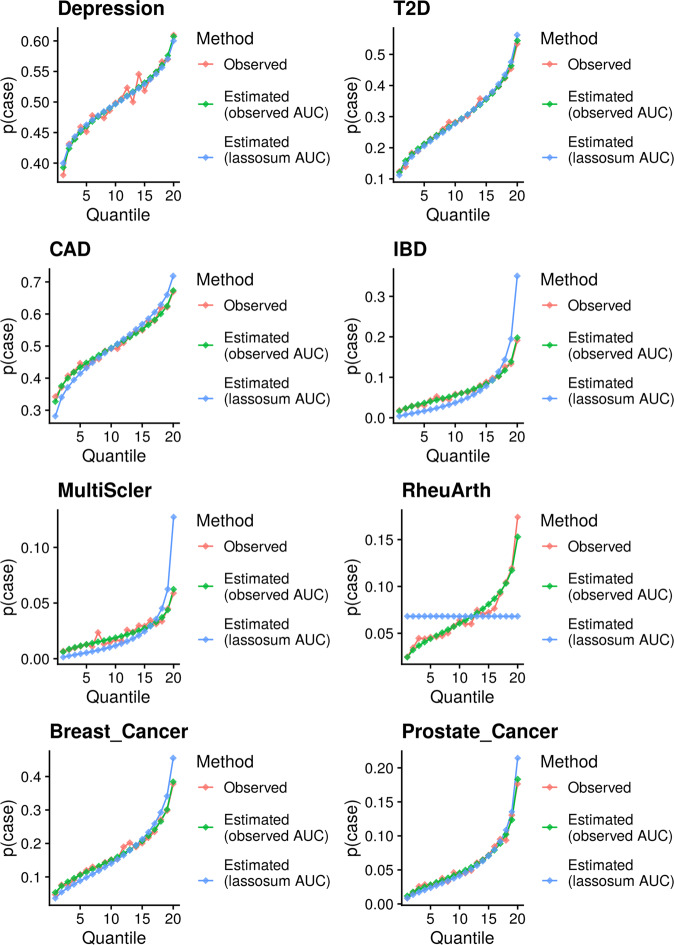
Comparison of observed and estimated probability of being a case across 20 DBSLMM polygenic score quantiles. Estimated values are based on either the observed polygenic score AUC, or the lassosum estimated AUC. Figures are available in colour online.

**Fig. 2:**
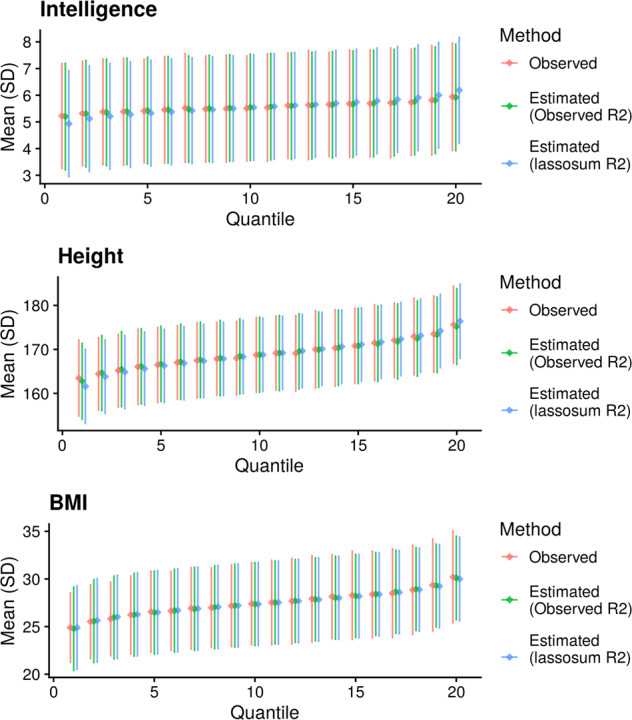
Comparison of observed and estimated phenotype mean and standard deviation across 20 DBSLMM polygenic score quantiles. Estimated values are either based on the observed polygenic score *R*^2^, or the lassosum estimated *R*^2^. Figures are available in colour online.

We validated the approach for converting a polygenic score into absolute terms by comparing the observed and estimated distributions of phenotypes within DBSLMM polygenic score quantiles. When the AUC/*R*^2^ of the polygenic score is known, the absolute estimates were highly concordant with observed values (Table 1 and Figs. 1 and 2).

For binary phenotypes (Table 1 and Fig. 1), the mean absolute difference between the observed and estimated proportion of cases was between 12.6% for MultiScler and 1.5% for both Depression and CAD. The concordance between observed and estimated values decreased as the number of cases within UKB decreased, reflecting increased error in the observed values.

For the three continuous phenotypes (Table 1 and Fig. 2), the mean absolute difference between observed and estimated means was <0.3%. The mean absolute difference between observed and estimated phenotype standard deviations across polygenic score quantiles were 1.3% for Intelligence, 1.5% for Height, and 6% for BMI. The reduced concordance between observed and estimated values for BMI reflects the increased skewness of this phenotype in UKB.

To demonstrate the flexibility of the conversion to model absolute risk within stratified populations, we compared observed and estimated absolute values for Height within males and females separately. Again, the concordance between observed and expected values was high (Fig. S1). Given large sex differences in Height, the polygenic score *R*^2^ increased when stratified by sex, and correspondingly the difference in mean Height across polygenic score quantiles was larger, and the standard deviation of Height within polygenic score quantiles was smaller.

The observed and estimated absolute values were also highly concordant when using pT + clump polygenic scores defined using stringent *p* value thresholds (Figs. S2 and S3). Some discrepancy between observed and estimated values was present for Depression due to the low predictive utility of the polygenic score when using the stringent *p* value threshold to select variants.

**Fig. 3:**
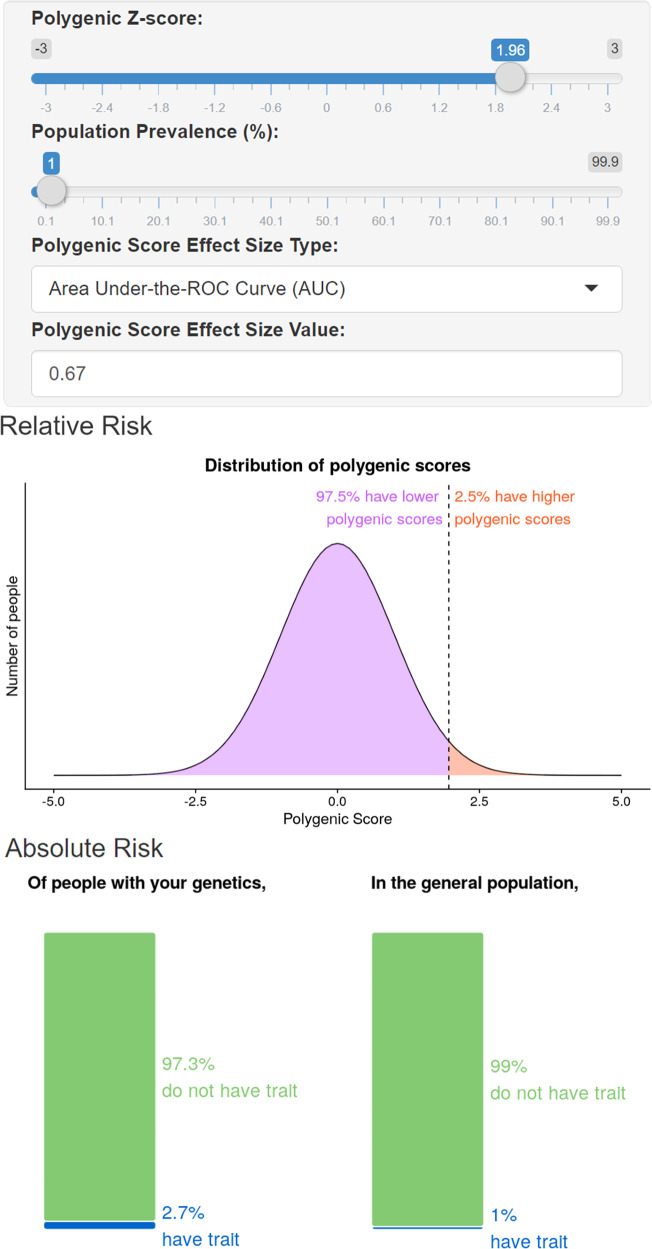
Shiny app implementing absolute scale conversion for binary phenotypes. Parameters reflect prevalence of schizophrenia and AUC of the schizophrenia polygenic score [36]. Figures are available in colour online.

**Fig. 4:**
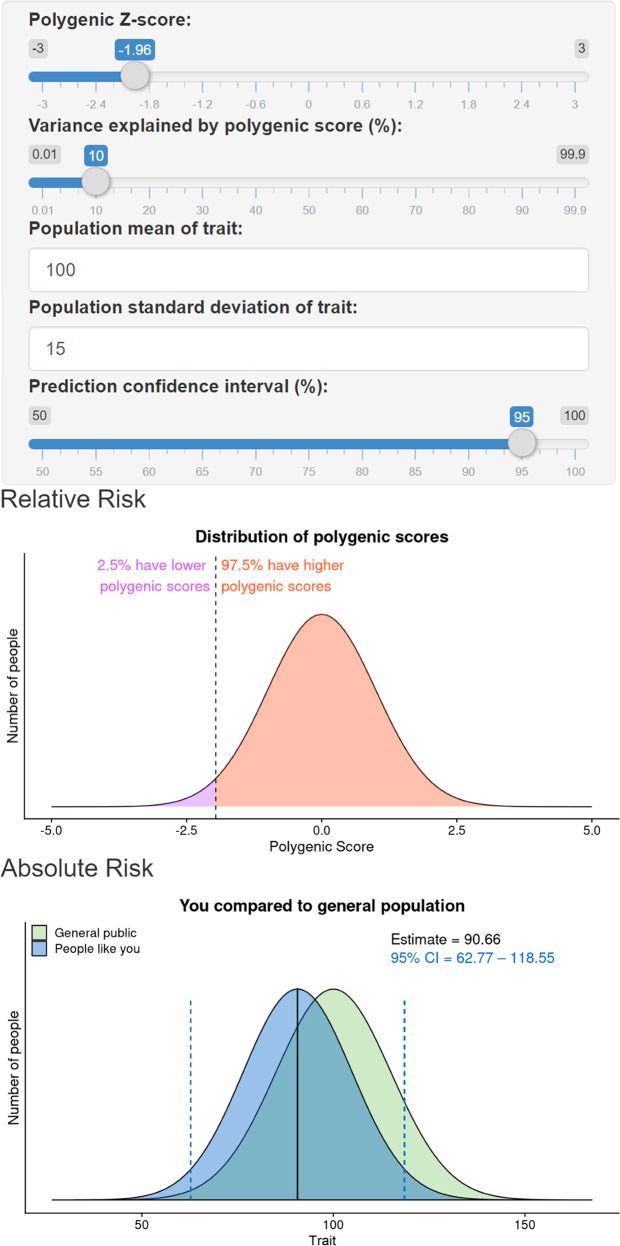
Shiny app implementing absolute scale conversion for normally distributed phenotypes. Parameters reflect mean and SD of IQ, and *R*^2^ of educational attainment polygenic score for IQ [37]. Figures are available in colour online.

We have provided examples using these conversions to interpret polygenic scores for an individual (Figs. 3 and 4). For binary phenotypes, we use the example schizophrenia, with a population prevalence of 1% and a polygenic score AUC of ~0.67 [36]. If an individual has a polygenic *Z*-score of 1.96, they are in the 97.5th percentile of schizophrenia polygenic scores. However, given the modest AUC of the polygenic score, only 2.7% of individuals with that polygenic score will develop schizophrenia (Fig. 3). For continuous phenotypes, we use the example of intelligence quotient (IQ), with a population mean of 100 and standard deviation of 15, for which the educational attainment polygenic score has been reported to explain 10% of the variance [37]. If an individual has a polygenic *Z*-score of −1.96, they are in the 2.5th percentile of educational attainment polygenic scores. The mean IQ of individuals with this polygenic score is 90.7, with 95% prediction intervals from 62.8 to 118.6 (Fig. 4).

To illustrate the full range of predictive utility of polygenic scores on the absolute scale, we have simulated results based on a range of polygenic score AUC and prevalence values for binary phenotypes, and range of polygenic scores *R*^2^ values for continuous phenotypes (Figs. S4 and S5).

### Validating polygenic scores AUC/*R*^2^ estimation

The lassosum estimates of AUC/*R*^2^ were concordant with the observed AUC/*R*^2^ of PRScs polygenic scores for most phenotypes (Table 2). The absolute difference between estimated and observed AUC values was less than 0.04 for five of the eight binary phenotypes. The absolute difference between estimated and observed *R*^2^ values were 0.007, 0.015, and 0.046 for BMI, Intelligence and Height, respectively. The lassosum estimates were most discordant for the three autoimmune disorders included in this study. The estimated AUC was substantially higher than the observed for IBD (AUC diff = 0.115) and MultiScler (AUC diff = 0.128). The analysis did not complete for RheuArth, resulting in an AUC of 0.5 being returned by the analysis.

**Table 2:** Comparison between polygenic score AUC/*R*^2^ observed in UKB and estimated using lassosum.

Binary			
Phenotype	Observed AUC	Estimated AUC	Difference
Depression	0.559	0.555	−0.004
T2D	0.642	0.651	0.010
CAD	0.597	0.624	0.027
IBD	0.677	0.792	0.115
MultiScler	0.657	0.785	0.128
RheuArth	0.632	0.500	−0.132
Breast_Cancer	0.658	0.698	0.040
Prostate_Cancer	0.691	0.721	0.030
Continuous			
Phenotype	Observed *R*^2^	Estimated *R*^2^	Difference
Intelligence	0.008	0.023	0.015
BMI	0.081	0.074	−0.007
Height	0.110	0.156	0.046

To determine the extent to which using estimated AUC/*R*^2^ influences the absolute estimates, we compared absolute estimates derived using lassosum estimated AUC/*R*^2^, to the observed absolute values (Table 3). The results for RheuArth were highly discordant due to the incomplete analysis estimating the AUC. After excluding RheuArth, the concordance between observed and estimated absolute values remained high when using estimated AUC/*R*^2^ values. For IBD and MultiScler, the phenotypes with discordant estimates of polygenic score AUC, the mean absolute percentage difference between observed-estimated proportion of cases was 38.6% and 43.7%, respectively. Discrepancies were particularly pronounced in the upper tail of the polygenic score distribution. The ratio between estimated and observed proportions of cases in the top polygenic score quantile (top 5%) was 1.83 and 2.16 for IBD and MultiScler, respectively.

**Table 3:** Comparison between observed and estimated case probabilities across polygenic score quantiles for binary phenotypes.

Phenotype	Mean Abs. Diff.	Mean Abs. Diff. of SD	*N*	*N*cas	*N*con	Skewness
Binary						
Depression	1.6%	NA	49,999	24,999	25,000	NA
T2D	3.4%	NA	49,999	14,888	35,111	NA
CAD	4.3%	NA	49,999	25,000	24,999	NA
IBD	38.6%	NA	49,999	3461	46,538	NA
MultiScler	43.7%	NA	49,999	1137	48,862	NA
RheuArth	40%	NA	49,999	3408	46,591	NA
Breast_Cancer	12.5%	NA	49,999	8512	41,487	NA
Prostate_Cancer	11.1%	NA	50,000	2927	47,073	NA
Continuous						
Intelligence	1.9%	1.5%	50,000	NA	NA	0.144
Height	0.2%	2.5%	49,999	NA	NA	0.117
BMI	0.3%	6%	49,999	NA	NA	0.592

Estimated values are based on the lassosum estimated AUC/R2 of the polygenic score.

*Mean Abs. Diff.* mean absolute difference between expected and observed case probability (binary) or trait mean (continuous), *Mean Abs. Diff. of SD* mean absolute difference between expected and observed trait standard deviation, *N* sample size, *Ncas* number of cases, *Ncon* number of controls.

## Discussion

This study has derived and evaluated methods for converting polygenic scores for both binary and normally distributed phenotypes from a relative value, of where the polygenic score lies on the distribution, into a value on the absolute scale. For a disorder, the conversion provides an estimate of the proportion of cases within a polygenic score quantile, and for a normally distributed trait, the conversion gives the trait mean and SD within a polygenic score quantile. Comparison of absolute estimates with observed values within UKB show the method is highly accurate when the AUC (for disorders) or *R*^2^ (for a trait) of the polygenic score is known. Furthermore, we show that lassosum pseudovalidate function can provide accurate estimates of AUC/*R*^2^ in most instances, though inaccuracies in AUC/*R*^2^ estimation can substantially bias absolute estimates in the extremes of the polygenic score distribution.

Interpretation of polygenic scores is one of the greatest barriers to their safe application to the clinical setting, and also poses a problem when polygenic scores are delivered to the individuals via direct-to-consumer genetics testing companies. Our study provides a summary statistic-based approach for converting polygenic scores into absolute terms, enabling their accurate interpretation. Although previous research has developed summary statistic-based approaches for converting polygenic scores to the absolute scale for binary outcomes [38], our study additionally provides a conversion method for normally distributed outcomes and a user-friendly webtool to improve accessibility of the methods. Furthermore, where the predictive utility of polygenic scores is unknown, we show that lassosum pseudovalidate may be used although the results should be interpreted with caution.

The predictive utility of polygenic scores is under a great deal of scrutiny. A major criticism is that they are only predictive at the group-level, i.e. for research, but are poor predictors at the individual-level. Our approach of converting polygenic scores to the absolute scale helps understand this distinction as it converts the group-level metric of predictive utility (AUC or *R*^2^) into an absolute value for an individual (risk of disorder or trait value with prediction intervals). Indeed, the absolute estimates do not vary substantially across the polygenic score distribution except at the extremes, which reflects the modest AUC/*R*^2^ of the current polygenic scores. The predictive utility of polygenic scores will increase with more powerful GWAS and better resolution of the causal variant. However, these scores will always be probabilistic, and will never give deterministic predictions as genetic variation only explains part of the phenotypic variance. Polygenic scores can be combined with other risk factors to increase the accuracy of prediction, though the value of prediction also depends partly on the actions or interventions available to address the predicted outcome.

Our approach focuses on converting polygenic scores onto the absolute scale given parameters describing the distribution of the outcome and PRS predictive utility in a representative sample. Therefore, our approach can be extended to account for other factors by modifying these input parameters. We demonstrate this flexibility by converting polygenic scores to the absolute scale for Height whilst accounting for sex differences by specifying the sex-specific mean and SD of Height, and the sex-specific PRS variance explained. This same approach could be used to model other factors modifying risk, such as smoking status for CAD. Of course, this summary statistic-based approach is limited by the availability of relevant summary statistics.

We have developed an interactive webtool implementing these methods to convert polygenic scores into absolute risk or trait estimates, also providing visual aids to promote the accurate interpretation of differences in risk. The webtool accepts any of the most used polygenic score effect size metrics, facilitating the use of resources such as the Polygenic Score Catalog [34], which collate polygenic score data and corresponding prediction metrics. We also plan to implement these methods on Impute.me, a popular non-profit website providing polygenic scores for users who upload genotype data from a direct-to-consumer genetic testing company.

There are several limitations of this study. Firstly, this summary statistic-based approach relies on the input parameters being representative of the target individual’s demographic. This study focuses on UKB participants of European ancestry, and we define the distribution of the phenotype using the observed distribution within UKB. However, specifying the distribution of a phenotype and the AUC/*R*^2^ of the polygenic score for a population that is representative of the target individual may be challenging. For example, assuming the phenotype distribution and polygenic score AUC/*R*^2^ found in a European population will give biased results in non-European populations where differences exist in the phenotype distribution and polygenic score AUC/*R*^2^ [39]. Most GWAS are performed in European populations, and the variance explained by polygenic scores is typically higher in Europeans than non-Europeans [40]. Secondly, although the lassosum pseudovalidate approach works well in most instances, it can provide inaccurate results, particularly for rare outcomes, which could lead to inaccurate absolute estimates. Further development of methods that can estimates the AUC/*R*^2^ of polygenic scores more reliably without a validation sample would be useful. Alternatively, the predictive utility of the polygenic score can be specified based on resources such as the Polygenic Score Catalog [34]. To support this effort, future polygenic score risk prediction studies should follow suggested reporting standards [41]. Thirdly, for continuous phenotypes, the approach is tailored to normally distributed phenotypes. Further development of methods to account for non-normally distributed phenotypes may be useful. Finally, for binary traits or disease, we calculate life-time risks that are based on pre-specified prevalence, which do not account for the risk period an individual has already lived through or other risk factors.

In summary, this study has provided an approach for converting polygenic scores into absolute risk and predictions based on GWAS summary statistics. It establishes a framework for appropriate and accurate interpretation of polygenic scores by patients, consumers, and healthcare professionals.

### URLs

- Interactive webtool/shiny apps: https://opain.github.io/GenoPred/PRS_to_Abs_tool.html
- LDSC HapMap 3 SNP-list: https://data.broadinstitute.org/alkesgroup/LDSCORE/w_hm3.snplist.bz2
- Impute.me: https://impute.me/
- GenoPred website: https://opain.github.io/GenoPred
- The Polygenic Score Catalog: https://www.pgscatalog.org/
- QCTOOL v2: https://www.well.ox.ac.uk/~gav/qctool_v2/index.html

## Supplementary information

Supplementary Text and Figures

Supplementary Table 1

Supplementary Table 2

Supplementary Table 3

## Data Availability

The data that support the findings of this study are available from the UK Biobank Access Management System (https://bbams.ndph.ox.ac.uk/ams/), but restrictions apply to the availability of these data, which were used under license for the current study, and so are not publicly available. For further information on how to access UKB data, see https://www.ukbiobank.ac.uk/enable-your-research/apply-for-access.
