# Supplementary material for: A tool for translating polygenic scores onto the absolute scale using summary statistics
Source: Eur J Hum Genet. 2022 Jan 4;30(3):339–48. doi: 10.1038/s41431-021-01028-z (PMC8904577; doi:10.1038/s41431-021-01028-z)
Supplement: Supplementary file 1 — Supplementary Text and Figures [file 41431_2021_1028_MOESM1_ESM.docx]

# Supplementary material for ‘**A Tool for Translating Polygenic Scores onto the Absolute Scale Using Summary Statistics’ by Pain et al.**

## UKB Outcome definitions

*Depression.* UKB participants were coded as depression cases if they met the Composite International Diagnostic Interview Short Form criteria for lifetime depression which was assessed in the online Mental Health Questionnaire (MHQ) using scoring protocols proposed by Davis et al (1). Depression cases were screened for indications of schizophrenia or bipolar disorder according to the MHQ. Controls excluded if they show any psychiatric indications according to the MHQ or depression indications according to: ICD-10 diagnoses; endorsement of self-reported depression; endorsement of current antidepressant usage; single or current depression according to the criteria adopted by Smith, et al (2). Further details of the exclusion criteria have been previously described (3).

*T2D.* Cases were identified based on a combination of hospital episode statistics, using both ICD-9 and ICD-10, the national death register, and self-reported questionnaire data. In order to classify as a case for type 2 diabetes, self-reported type 2 or generic diabetes status was established in the nurse interview and the touchscreen questionnaire. However, participants were only classified as cases when they reported in the questionnaire that they had not been treated with insulin in the first year after diagnosis and had been diagnosed after the age of 35 years. Type 2 diabetes controls did not fulfil these criteria and did not have any other types of diabetes. Further details of the T2D definition have been previously published (4).

*Coronary artery disease (CAD).* Participants who were registered in the hospital in-patient data or the death register to have had ischemic heart diseases, or participants who had coronary revascularization operations were classified as coronary artery disease cases in this study. If participants self-reported those conditions in the nurse interview or the touchscreen questionnaire, they were also considered to have coronary artery disease. Coronary artery disease controls did not fulfil those criteria. Further details of the CAD definition have been previously published (4).

*Autoimmune diseases (IBD, RheuArth, MultiScler):* UKB participants were coded as autoimmune cases if at least two of the following measures were observed: ICD-10 diagnoses from Hospital Episode Statistics; endorsement of self-reported autoimmune diseases; endorsement of prescription medication for the corresponding autoimmune diseases. More than one hospital admission for the respective autoimmune conditions was also sufficient. Controls were excluded if any of the following were observed: Pernicious Anemia, Autoimmune Thyroid Disease, Type 1 diabetes, Multiple Sclerosis, Myasthenia Gravis, Coeliac, Inflammatory Bowel Disease, Hidradenitis Suppurativa, Pemphigoid/Pemphigus, Psoriasis, Ankylosing Spondylitis, Polymyalgia Rheumatica/Giant Cell Arteritis, Psoriatic Arthritis, Rheumatoid Arthritis, Sjögren Syndrome, Systemic Lupus Erythematosus.

*Intelligence* was defined using the Fluid intelligence score variable. Fluid intelligence was assessed using the 13 item UKB Touch-screen Fluid intelligence test (5). The test measures the capacity to solve problems that require logic and reasoning ability, independent of acquired knowledge. The fluid intelligence variable was derived by UKB as an unweighted sum of the number of correct answers, assigning a score of 0 to unanswered questions.

*Height* was defined using the Standing height variable (Field ID: f.50.0.0).

*BMI* was defined using the Body mass index variable (Field ID: f.21001.0.0).

Breast Cancer and Prostate Cancer were defined using the self-reported illness codes (1044 = prostate cancer, 1002 = breast cancer, Field ID: f.20001).

## Converting relative estimates into absolute terms

### Binary traits

**Aim:** We wish to estimate the risk of disease for an individual given their polygenic score falls within a certain group of the population distribution, defined by quantiles, using only summary statistic data; specifically, the disease prevalence in the population understudy and the AUC (area under the ROC curve, here measuring how useful the polygenic score is at classifying individuals as cases or controls).

We achieve this aim in three steps. Firstly, we use the summary statistics to define the distribution of the polygenic score in the case subpopulation, the control subpopulation and the overall population. Secondly, we use the distribution of the polygenic score in the population to define the quantiles (the cut points defining the groups). Thirdly, we use the polygenic score distribution in the case (control) subpopulation to derive the required probability (the probability of being a case or control given the polygenic score falls within a certain group).

**Step 1: Defining the conditional and unconditional polygenic score distributions**

Let $Y_{i}$ denote the random disease outcome variable for individual $i$ such that $Y_{i}=1$ if the individual is a case and $Y_{i}=0$ if the individual is a control. The distribution of $Y_{i}$ is defined as:

$$Y_{i}\sim Binom\left( 1, K \right)$$

where $K=p\left( Y_{i}=1 \right)$ is the disease prevalence.

Let $X_{i}$ denote the random polygenic score variable for individual $i$. If individual $i$ is a control, then we assume that their polygenic score follows a standard normal distribution:

$$X_{i}|\left\{ Y_{i}=0 \right\}\sim N\left( 0, 1 \right)$$

If individual $i$ is a case, then we assume that their polygenic score is also normally distributed but with a different mean:

$$X_{i}|\left\{ Y_{i}=1 \right\}\sim N\left( d, 1 \right)$$

where $d$ is Cohen’s *d*, which measures the standardised difference between two means (here it is the standardised difference between the expected polygenic score for cases and controls).

Then, the distribution for $X_{i}$ is a *mixture* of the above two normal distributions, weighted by the disease prevalence, with the probability density function (PDF) for $X_{i}$ defined as:

$$f_{X_{i}}\left( x_{i} \right)=Kf_{X_{i}|Y_{i}}\left( x_{i}|1 \right)+(1-K)f_{X_{i}|Y_{i}}\left( x_{i}|0 \right)$$

where $f_{X_{i}|Y_{i}}\left( x_{i1}|1 \right)=\frac{1}{\sqrt{2\pi}}e^{-\frac{1}{2}{(x_{i}-d)}^{2}}$ is the PDF for the conditional polygenic score random variable given the individual is case ($X_{i}|\left\{ Y_{i}=1 \right\}$).

Similarly, $f_{X_{i}|Y_{i}}\left( x_{i}|0 \right)=\frac{1}{\sqrt{2\pi}}e^{-\frac{1}{2}{x_{i}}^{2}}$ is the PDF for the conditional polygenic score random variable given the individual is a control ($X_{i}|\left\{ Y_{i}=0 \right\}$).

Rice and Harris (6) showed that Cohen’s *d* can be approximated using:

$$d\approx\sqrt{2}\Phi^{-1}[AUC]$$

Therefore, the above distributions for the polygenic score can be defined using the AUC and the disease prevalence.

**Step 2: Defining the quantiles**

To define the required polygenic score groups we need to calculate the quantiles; the $(n-1)$ cut points that split the data into $n$ equally sized groups. The quantiles are defined in the overall population encompassing both cases and controls and we therefore need to use to unconditional polygenic score distribution (which is a mixture of two normal distributions).

Let:

- $p_{q}=q/n$ be the probability value defining a quantile boundary, and,
- $t_{q}$ be the polygenic score value corresponding to $p_{q}$;

such that:

$$p_{q}=p\left( X_{i}<t_{q} \right)$$

for $q=1,\cdots,n-1$.

Since $X_{i}$ is a mixture of two normal distributions, this becomes:

$$p_{q}=Kp\left( X_{i} | \left\{ Y_{i}=1 \right\}< t_{q} \right)+\left( 1-K \right)p\left( X_{i} | \left\{ Y_{i}=0 \right\}< t_{q} \right)$$

$$=Kp\left( Z<t_{q}-d \right)+\left( 1-K \right)p\left( Z<t_{q} \right)$$

$$=K\Phi\left[ t_{q}-d \right]+\left( 1-K \right)\Phi[t_{q}]$$

Variables $p_{q}$, $K$ and $d$ are known. The only unknown in the above equation is $t_{q}$, which can be found by solving this equation using numerical methods. For example, we used the uniroot function within R, which finds the root (solution) of an equation by searching a specified interval for the parameter that is required to be calculated and outputting the value that gives a 0 solution to the equation. The user therefore needs to input the equation such that it equals 0. In this case, that is:

$$K\Phi\left[ t_{q}-d \right]+\left( 1-K \right)\Phi\left[ t_{q} \right]-p_{q}=0$$

This will need to be repeated to calculate all quantiles.

**Step 3: Equations for the required case and control probabilities**

We now wish to derive the probability that individual $i$ is a case or a control given they have a polygenic score within a certain range (defined by the quantiles). Starting with the probability of being a case, we wish to calculate:

$$p(Y_{i}=1|t_{q-1}<X_{i}<t_{q})$$

for $q=1,\ldots,21$, where: 1. $t_{0}=-\infty$, 2. $t_{21}=\infty$, and 3. the remaining values of $t_{q-1}$ and $t_{q}$ are calculated using step 2.

Using rules of conditional probability we write:

$$p\left( Y_{i}=1 | t_{q-1}<X_{i}<t_{q} \right)=\frac{p\left( t_{q-1}<X_{i}<t_{q} | Y_{i}=1 \right)p\left( Y_{i}=1 \right)}{p\left( t_{q-1}<X_{i}<t_{q} \right)}$$

$t_{q-1}$ and $t_{q}$ define consecutive quantiles. Therefore $p\left( t_{q-1}<X_{i}<t_{q} \right)=1/n$ is a constant value for all $q=1, \ldots, (n+1)$, where $n$ is the number of groups the quantiles define. Additionally, $p\left( Y_{i}=1 \right)=K$. Therefore:

$$p\left( Y_{i}=1 | t_{q-1}<X_{i}<t_{q} \right)=nK p\left( t_{q-1}<X_{i}<t_{q} | Y_{i}=1 \right)$$

Recall that $E\left[ X_{i} | Y_{i}=1 \right]=d$, and so:

|  | $p\left( Y_{i}=1 \vert t_{q-1}<X_{i}<t_{q} \right)=nK\left( p\left( Z<t_{q}-d \right)-p\left( Z<t_{q-1}-d \right) \right)$ $=nK\left( \Phi\left[ t_{q}-d \right]-\Phi\left[ t_{q-1}-d \right] \right)$ | **Eq 1** |
| --- | --- | --- |

where $Z\sim N(0,1)$ and:

$$\Phi\left[ x \right]=\int_{-\infty}^{x} \frac{1}{\sqrt{2\pi}}e^{-\frac{1}{2}s^{2}}ds$$

is the cumulative distribution function (CDF) for the standard normal distribution.

The probability of being a control given the polygenic score sits within a certain range (defined by the quantiles) is derived in a similar way giving:

|  | $p\left( Y_{i}=0 \vert t_{q-1}<X_{i}<t_{q} \right)=nK\left( \Phi\left[ t_{q} \right]-\Phi\left[ t_{q-1} \right] \right)$ | **Eq 2** |
| --- | --- | --- |

The probabilities given in *Eq 1* and *Eq 2* can be used absolute risks, or to calculate further summary measures such as quantile relative risks or odds ratios.

Note**:** In the above run-through, where the distributions of polygenic scores conditional on disease status are both standardised to have a variance of 1, the expectation and variance of the polygenic score in the overall population are:

$$\mu_{X}=E\left[ X_{i} \right]=dK$$

And:

$$\sigma_{X}^{2}=Var\left[ X_{i} \right]=1+K(1-K)d^{2}$$

However, typically results are presented such that the overall distribution of the polygenic score is centred and standardised to unit variance and this is what is used in the graphical presentation of results from the R-shiny developed here. This standardisation means the distribution of the polygenic score within cases is:

$$Z_{PRS,i}|\{Y_{i}=1\}\sim N\left( \frac{d-\mu_{X}}{\sigma_{X}}, \frac{1}{\sigma_{X}^{2}} \right)$$

and within controls is:

$$Z_{PRS,i}|\{Y_{i}=0\}\sim N\left( \frac{-\mu_{X}}{\sigma_{X}}, \frac{1}{\sigma_{X}^{2}} \right)$$

thereby giving $E\left[ Z_{PRS,i} \right]=0$ and $Var\left[ Z_{PRS,i} \right]=1$.

### Normally distributed traits

**Aim:** To estimate the mean (and variance) of the outcome trait for individuals with polygenic score within a group, defined by quantiles, using only summary statistics. The summary statistic used here is the variance in outcome explained by the polygenic score, $R^{2}$.

To achieve this aim we need to: 1. define the joint distribution of the outcome trait and the polygenic score, 2. calculate the quantiles, and 3. use this joint distribution and the quantiles to estimate the required distribution parameters for outcome conditional on the polygenic score belonging to a given group.

**Step 1: Defining the joint distribution of the outcome trait and the polygenic score**

For simplicity, let us assume that the outcome trait, $Y_{i}$, and the polygenic score, $X_{i}$, are standardised and follow a bivariate normal distribution defined as:

$$\left[ \begin{matrix} Y_{i} \\ X_{i} \end{matrix} \right]\sim N\left( \underline{\mu}, \Sigma\right)$$

where $\underline{\mu}=\left[ \begin{matrix} 0 \\ 0 \end{matrix} \right], \Sigma=\left[ \begin{matrix} 1 & R \\ R & 1 \end{matrix} \right]$ and $R^{2}$ is the variance in outcome explained by the polygenic score.

**Step 2: Defining the quantiles**

Recall:

$$p_{q}=p\left( X_{i}<t_{q} \right)=\frac{q}{n}$$

where:

- $p_{q}$ is the probability value defining a quantile (cut point) such that the data is split into $n$ equally sized groups, and,
- $t_{q}$ is the polygenic score value corresponding to $p_{q}$.

Here, polygenic score quantile boundaries are calculated using the univariate polygenic score distribution: $X_{i}\sim N\left( 0,1 \right)$. Therefore:

$$p_{q}=\Phi\left[ t_{q} \right]$$

and:

$$t_{q}=\Phi^{-1}\left[ p_{q} \right]$$

for $q=1,\ldots, n-1$.

**Step 3: Estimating the expected value of the outcome trait given the polygenic score falls within a quantile**

The principal value of interest is the expected value of the outcome trait given the polygenic score belongs to a certain quantile; that is, $E[Y_{i}|{\{t}_{q-1}<X_{i}<t_{q}\}]$.

To find this we need to solve the following:

$$E[Y_{i}|{\{t}_{q-1}<X_{i}<t_{q}\}]=n\int_{-\infty}^{+\infty} y\int_{t_{q-1}}^{t_{q}} f_{Y_{i},X_{i}}\left( y,x \right)dxdy$$

where:

$$f_{Y_{i},X_{i}}\left( y,x \right)=\frac{1}{\sqrt{\left( 2\pi\right)^{2}\left| \Sigma\right|}}exp\left( -\frac{1}{2}\left[ \begin{matrix} y & x \end{matrix} \right]\Sigma^{-1}\left[ \begin{matrix} y \\ x \end{matrix} \right] \right)$$

$$=\frac{1}{2\pi\sqrt{1-R^{2}}}exp\left( -\frac{1}{2(1-R^{2})}\left( y^{2}-2Rxy+x^{2} \right) \right)$$

is the joint (bivariate normal) probability distribution function for the outcome trait random variable ($Y_{i}$) and the polygenic score random variable ($X_{i}$). $\left| \Sigma\right|$ is the determinant of the covariance matrix $\Sigma$.

Solving such an integral is done using numerical methods. Here we use the ‘mtmvnorm’ function in the ‘tmvtnorm’ R package (7), which outputs:

$$\underline{\mu}^{'}=\left[ \begin{matrix} E[Y_{i}|{\{t}_{q-1}<X_{i}<t_{q}\}] \\ E[X_{i}|{\{t}_{q-1}<X_{i}<t_{q}\}] \end{matrix} \right]$$

and

$$\Sigma^{'}=\left[ \begin{matrix} Var[Y_{i}|{\{t}_{q-1}<X_{i}<t_{q}\}] & Cov\left[ Y_{i} | {\{t}_{q-1}<X_{i}<t_{q} \right\},X_{i}|{\{t}_{q-1}<X_{i}<t_{q}\}] \\ Cov\left[ Y_{i} | {\{t}_{q-1}<X_{i}<t_{q} \right\},X_{i}|{\{t}_{q-1}<X_{i}<t_{q}\}] & Var[X_{i}|{\{t}_{q-1}<X_{i}<t_{q}\}] \end{matrix} \right]$$

## Conversion of *R* into Cohen’s *d*

The Cohen’s *d* is calculated from *R* as:

|  | $d=\frac{\sqrt{a}\times R}{\sqrt{1-R^{2}}}$ | (1) |  |
| --- | --- | --- | --- |

where $a$ is a correction factor for imbalanced GWAS sampling ratio ($n_{1}\neq n_{2}$),

|  | $a=\frac{\left( n_{1}+n_{2} \right)^{2}}{n_{1}\times n_{2}}$ | (2) |  |
| --- | --- | --- | --- |

The AUC is calculated from Cohen’s *d* as:

|  | $AUC=Ф\left[ \frac{d}{\sqrt{2}} \right]$ | (3) |  |
| --- | --- | --- | --- |

where $Ф$ is the normal cumulative distribution function.

## Additional approach explored for estimation of polygenic score AUC/R2

### AVENGEME/LDSC

The ‘AVENGEME’ R package (8) includes a function called ‘polygenescore’ which has several purposes including the estimation of the AUC/*R*^2^ of polygenic scores derived using the pT+clump approach. AVENGEME requires various input parameters, including the SNP-based heritability of the phenotype and the proportion of variants with zero effect (pi0; inverse of polygenicity). The SNP-based heritability was estimated from the GWAS summary statistics using LD-score regression (LDSC) (9). However, pi0 is challenging to estimate from GWAS summary statistics, and so a range of pi0 values were used (0.92, 0.94, 0.96, 0.98). As a sensitivity analysis, AVENGEME analysis was also performed using the SNP-based heritability estimated by AVENGEME’s ‘estimatePolygenicModel’, using the observed pT+clump polygenic score associations.

The AVENGEME estimated AUC/*R*^2^ of polygenic scores based on LDSC and AVENGEME SNP-based heritability and a range of pi0 parameters are shown in Figure S6-S7. The figures show the observed AUC/*R*^2^ of pT+clump polygenic scores across a range of p-value thresholds, and the observed AUC/*R*^2^ of DBSLMM polygenic scores. The results show that AVENGEME estimates of AUC/*R*^2^ are not affected by the pi0 parameter when the p-value threshold (pT) is equal to 1. However, pT=1 is often not the optimal pT, particularly for less polygenic phenotypes, and therefore using the AVENGEME estimate of AUC/*R*^2^ based on a pT=1 can lead to an underestimation of AUC/*R*^2^. There are two important observations from the results.

First, the accuracy of AVENGEME estimates of AUC/*R*^2^ is influenced by LDSC SNP-based heritability estimates. For example, LDSC SNP-based heritability for MultiScler is low, leading to low estimates of AUC/*R*^2^ by AVENGEME (Table S1). However, when the SNP-based heritability of MultiScler is estimated by AVENGEME using the observed pT+clump associations, the SNP-based heritability is higher, leading to an increased AUC/*R*^2^ estimate. The discrepancy in SNP-based heritability for MultiScler between LDSC and AVENGEME is interesting, with previous literature suggest the LDSC estimate is downward biased (10). However, it is the discordance between the two methods that leads to in accurate estimates of AUC/*R*^2^, highlighting a limitation of this approach.

Second, AVENGEME is designed to model pT+clump polygenic scores and is therefore not well suited to polygenic scores derived using more modern shrinkage-based polygenic scoring methods, such as DBSLMM. The results show that the estimated AUC/*R*^2^ of polygenic scores for the best pT and a pi0 parameter of 0.94 is similar to the observed AUC/*R*^2^ of the DBSLMM polygenic score. However, assuming a pi0 parameter of 0.94 does not allow for differences in polygenicity across phenotypes, leading to underestimation of AUC/*R*^2^ for low polygenicity phenotypes, and overestimation of AUC/*R*^2^ for high polygenicity outcomes (Table S3-S4).

These results indicate the AVENGEME/LDSC approach is not well suited to estimation of AUC/*R*^2^ for polygenic scores derived using approaches such as DBSLMM based on summary statistics alone.

### G-WIS

We also explored the G-WIZ R package (11). This method is able to estimate the AUC of polygenic scores derived from GWAS of binary outcomes, but it cannot currently be applied to continuous outcomes. A brief investigation of the method also highlighted that substantial computational resources would be required to model polygenic scores derived using genome-wide variation, as opposed to a smaller number of genome-wide significant variants. For these reasons, we have not considered this approach further.

## Converting between an odds ratio and Cohen’s *d*

### Aim

Given the disease prevalence within the *study* population ($K_{s}$), we aim to derive an equation to transform the odds ratio for a standardised polygenic score (${OR}_{X^{'}}$) to Cohen’s *d* ($d$). Here, $d$ is the standardised mean difference between the polygenic score for cases and the polygenic score for controls.

Conversion

$$d\sqrt{1+d^{2}K_{s}\left( 1-K_{s} \right)}=\log({OR}_{X^{'}})$$

### Methods

#### Distribution assumptions in the study population

Let $Y$ denote a binary disease trait random variable and let $S$ denote a sampling random variable, which will equal 1 if an individual is included in the study population and 0 otherwise. The conditional distribution of disease outcome for an individual $i$ given they are sampled is given by:

$$Y_{i}|\left\{ S_{i}=1 \right\} \sim Bi(1,K_{s})$$

where $K_{s}=p\left( Y_{i}=1 | S_{i}=1 \right)$ is the probability of being a case in the study population. This is not necessarily the same as the prevalence of disease in general population ($K=p(Y_{i}=1)$). We assume that sampling only depends on disease status.

Let $X_{i}$ denote the polygenic score random variable for individual $i$. The distribution of this polygenic score variable depends on the disease status for the individual. Here we define these conditional distributions for $X_{i}$ as:

|  | $X_{i}\vert\left\{ Y_{i}=1 \right\} \sim N(d, 1)$ | (1) |
| --- | --- | --- |

and:

|  | $X_{i}\vert\left\{ Y_{i}=0 \right\} \sim N(0, 1)$ | (2) |
| --- | --- | --- |

where $d$ is Cohen’s *d* (the standardised difference between two means). The above assumes that the variance of the polygenic score in cases equals the variance of the polygenic score in controls.

We assume that the distribution of a polygenic score, $X_{i}$, is independent of sampling given disease status:

$$X_{i}\perp S_{i}|Y_{i}$$

Therefore within the study population:

|  | $X_{i}\vert\left\{ Y_{i}=1, S_{i}=1 \right\}= X_{i}\vert\left\{ Y_{i}=1 \right\} \sim N(d, 1)$ |  |
| --- | --- | --- |

and:

|  | $X_{i}\vert\left\{ Y_{i}=0, S_{i}=1 \right\}= X_{i}\vert\left\{ Y_{i}=0 \right\} \sim N(0, 1)$ |  |
| --- | --- | --- |

as above.

The distribution for the polygenic score within the *study population* is a mixture of the above two distributions weighted by the *sample* disease probability, $K_{s}$. The probability distribution function (PDF) for $X_{i}|\{S_{i}=1\}$ is then given by:

$$f_{X_{i}|S_{i}}\left( x_{i}|s_{i}=1 \right)= {K_{s}f}_{X_{i}|Y_{i}}\left( x_{i} | y_{i}=1 \right)+ \left( 1-K_{s} \right)f_{X_{i}|Y_{i}}\left( x_{i} | y_{i}=0 \right)$$

where $f_{X_{i}|Y_{i}}\left( x_{i} | y_{i} \right)$ is the conditional PDF for the polygenic score given disease status, corresponding to the normal distribution in Equation (1) when individual $i$ is a case ($y_{i}=1$) and Equation (2) when individual $i$ is a control ($y_{i}=0$).

The mean and variance $X_{i}|\{S_{i}=1\}$ are then given by:

|  | $\mu_{X_{i}\vert S_{i}=1}=dK_{s}$ | (3) |
| --- | --- | --- |

and:

|  | $\sigma_{X_{i}\vert S_{i}=1}^{2}=1+d^{2}K_{s}\left( 1-K_{s} \right)$ | (4) |
| --- | --- | --- |

respectively.

#### Odds ratio definition 1

Using a logistic model, we can write the following relationship between the probability of disease and an observed polygenic score for individual $i$ within the study population:

$$\log\left( \frac{p(Y_{i}=1|X_{i}=x_{i}, S_{i}=1)}{p(Y_{i}=0|X_{i}=x_{i}, S_{i}=1)} \right)=\beta_{0}+\beta_{1}x_{i}$$

The odds ratio (OR) is then defined as:

$$OR= \frac{p(Y_{i}=1|X_{i}=x_{i}+1)}{p(Y_{i}=0|X_{i}=x_{i}+1)}\frac{p(Y_{i}=0|X_{i}=x_{i})}{p(Y_{i}=1|X_{i}=x_{i})}=e^{\beta_{1}}$$

Note, the sampling variable (and so study population case-control ratio) is captured through the intercept here, and therefore the OR does not depend on $S_{i}$.

Using conditional probability, we can re-write this OR as a function of Cohen’s *d* using the distributions defined in Equations (1) and (2). That is:

$$OR= \frac{p\left( X_{i}=x_{i}+1 | Y_{i}=1 \right)}{p\left( X_{i}=x_{i}+1 | Y_{i}=0 \right)}\frac{p\left( X_{i}=x_{i} | Y_{i}=0 \right)}{p\left( X_{i}=x_{i} | Y_{i}=1 \right)}$$

$$= \frac{f_{X_{i}|Y_{i}}\left( x_{i}+1 | y_{i}=1 \right)}{f_{X_{i}|Y_{i}}\left( x_{i}+1 | y_{i}=0 \right)}\frac{f_{X_{i}|Y_{i}}\left( x_{i} | y_{i}=0 \right)}{f_{X_{i}|Y_{i}}\left( x_{i} | y_{i}=1 \right)}$$

$$=\frac{exp\left( -\frac{1}{2}\left( x_{i}+1-d \right)^{2} \right)}{exp\left( -\frac{1}{2}\left( x_{i}+1 \right)^{2} \right)}\frac{exp\left( -\frac{1}{2}\left( x_{i} \right)^{2} \right)}{exp\left( -\frac{1}{2}\left( x_{i}-d \right)^{2} \right)}$$

Multiplying out the squares reveals that:

$$OR=exp(d)$$

Therefore:

|  | $d=\beta_{1}$ | (5) |
| --- | --- | --- |

This OR corresponds to the conditional risk of disease given $\{X_{i}=x_{i}\}$. However, ORs provided in the literature tend to be standardised such that the variance of the unconditional polygenic score *within* the study population equals 1, where $X_{i}|\{S_{i}=1\}$ has mean and variance given in Equations (3) and (4).

#### Odds ratio definition 2

Let us define the standardised polygenic score random variable for individual $i$ as:

$$X_{i}^{'}=\frac{X_{i}-\mu_{X_{i}|S_{i}=1}}{\sigma_{X_{i}|S_{i}=1}}=\frac{X_{i}-dK_{s}}{\sqrt{1+d^{2}K_{s}(1-K_{s})}}$$

Then, the logistic model relating the risk of disease to this standardised polygenic score is given by:

$$\log\left( \frac{p\left( Y_{i}=1 | X_{i}^{'}=x_{i}^{'}, S_{i}=1 \right)}{p\left( Y_{i}=0 | X_{i}^{'}=x_{i}^{'}, S_{i}=1 \right)} \right)=\beta_{0}^{'}+\beta_{1}^{'}x_{i}^{'}$$

$$=\beta_{0}^{'}+\beta_{1}^{'}\left( \frac{x_{i}-\mu_{X_{i}|S_{i}=1}}{\sigma_{X_{i}|S_{i}=1}} \right)$$

$$=\beta_{0}^{'}-\frac{\mu_{X_{i}|S_{i}=1}}{\sigma_{X_{i}|S_{i}=1}}+\frac{\beta_{1}^{'}}{\sigma_{X_{i}|S_{i}=1}}x_{i}$$

$$=\beta_{0}+\beta_{1}x_{i}$$

$$=log \left( \frac{p(Y_{i}=1|X_{i}=x_{i}, S_{i}=1)}{p(Y_{i}=0|X_{i}=x_{i}, S_{i}=1)} \right)$$

Using this, and Equations (4) and (5), we can write:

$$d=\beta_{1}=\frac{\beta_{1}^{'}}{\sigma_{X_{i}|S_{i}=1}}=\frac{\beta_{1}^{'}}{\sqrt{1+d^{2}K_{s}\left( 1-K_{s} \right)}}$$

where $\beta_{1}^{'}=\log({OR}_{X^{'}})$, with ${OR}_{X^{'}}$ being the odds ratio typically reported in the literature, corresponding to a within-sample standardised polygenic score.

Therefore:

$$d\sqrt{1+d^{2}K_{s}\left( 1-K_{s} \right)}=\log({OR}_{X^{'}})$$

With this equation, for a given $K_{s}$, the odds ratio can be calculated using Cohen’s *d* and vice versa. We note, to obtain an estimate of Cohen’s *d* using the odds ratio this equation should be used within a root finding function, such as uniroot within R.

# GWAS summary statistic quality control

GWAS summary statistics underwent quality control to extract HapMap3 variants, remove ambiguous variants, remove variants with missing data, flip variants to match the reference retain variants with a minor allele frequency (MAF) > 0.01 in the European subset of 1KG Phase 3, retain variants with a MAF > 0.01 in the GWAS sample (if available), retain variants with a INFO > 0.6 (if available), remove variants with a discordant MAF (>0.2) between the reference and GWAS sample (if available), remove variants with association p-values >1 or </=0, remove duplicate variants, and remove variants with sample size >3SD from the median sample size (if per variant sample size is available). The GWAS quality control criteria are in-line with other software. For example, the LDSC munge_sumstats.py script removes variants with p-values >1 or <=0 (see URLs), and SBayesR filters variants with a sample size 3SD from the median (12).


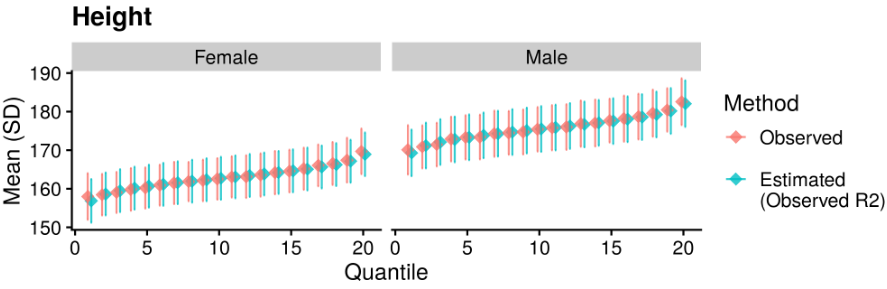


Figure S1. Validation of conversion to absolute scale using height stratified by sex. Compares observed and estimated phenotype mean and standard deviation across 20 polygenic score quantiles. Estimated values are based on the observed polygenic score R^2^ within males and females.


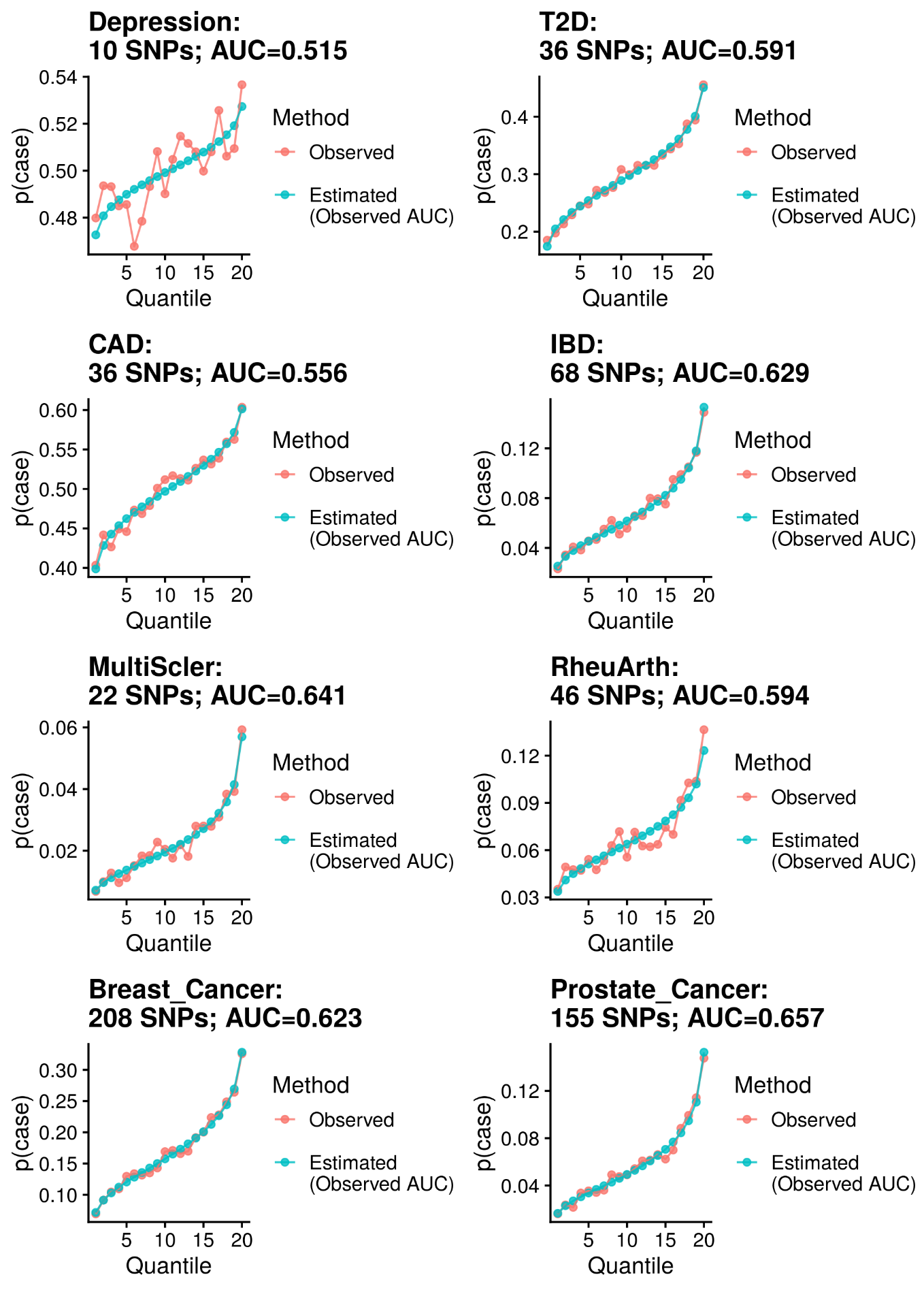


Figure S2. Comparison of observed and estimated probability of being a case across 20 pT+clump polygenic score quantiles. The pT+clump polygenic scores are derived using the most stringent p-value threshold retaining at least 5 variants. Estimated values are based on the observed polygenic score AUC. The number of SNPs considered in the polygenic score, and the AUC of the polygenic score are shown for each phenotype.


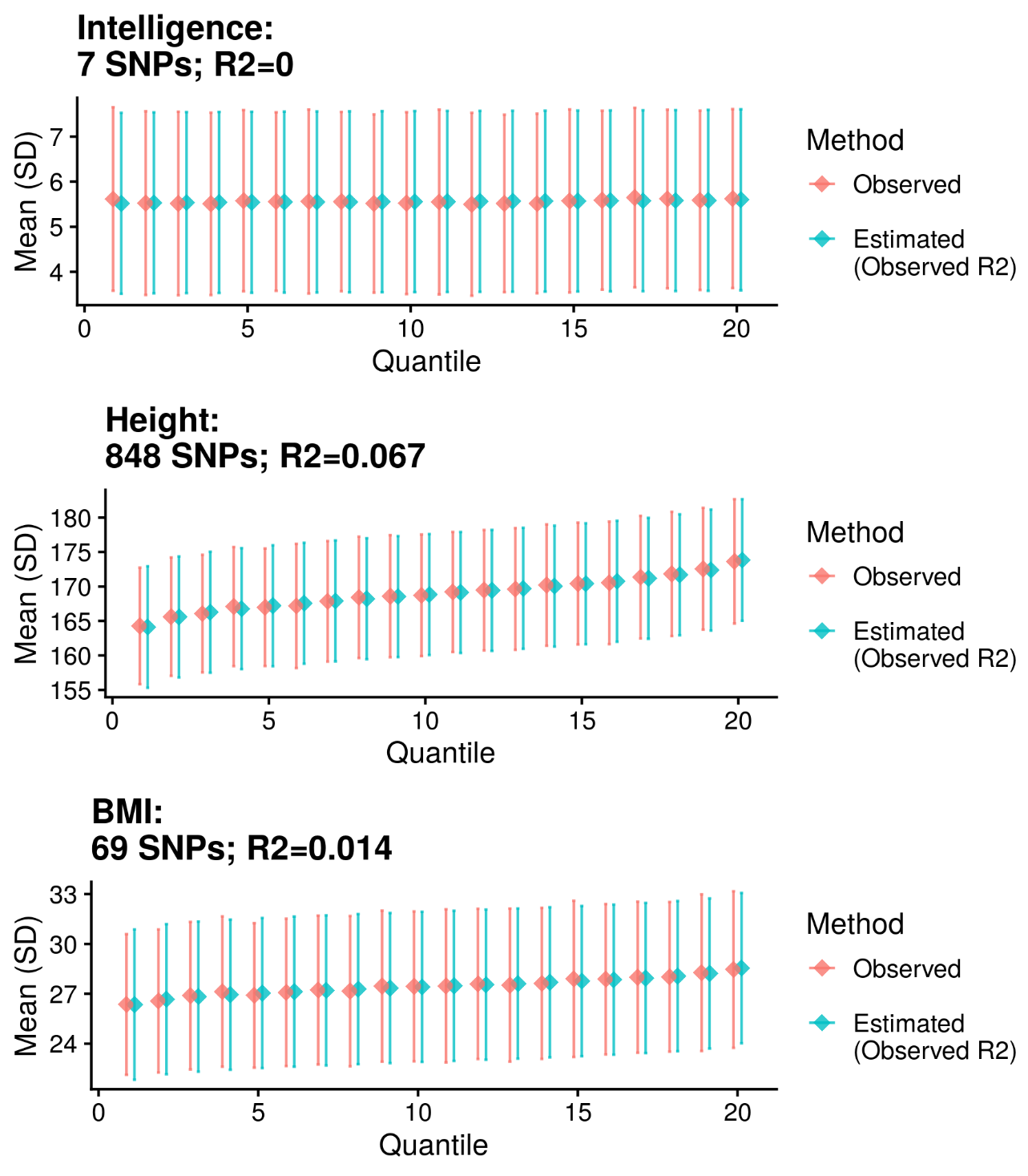


Figure S3. Comparison of observed and estimated phenotype mean and standard deviation across 20 pT+clump polygenic score quantiles. The pT+clump polygenic scores are derived using the most stringent p-value threshold retaining at least 5 variants. Estimated values are based on the observed polygenic score R^2^. The number of SNPs considered in the polygenic score, and the R^2^ of the polygenic score are shown for each phenotype.


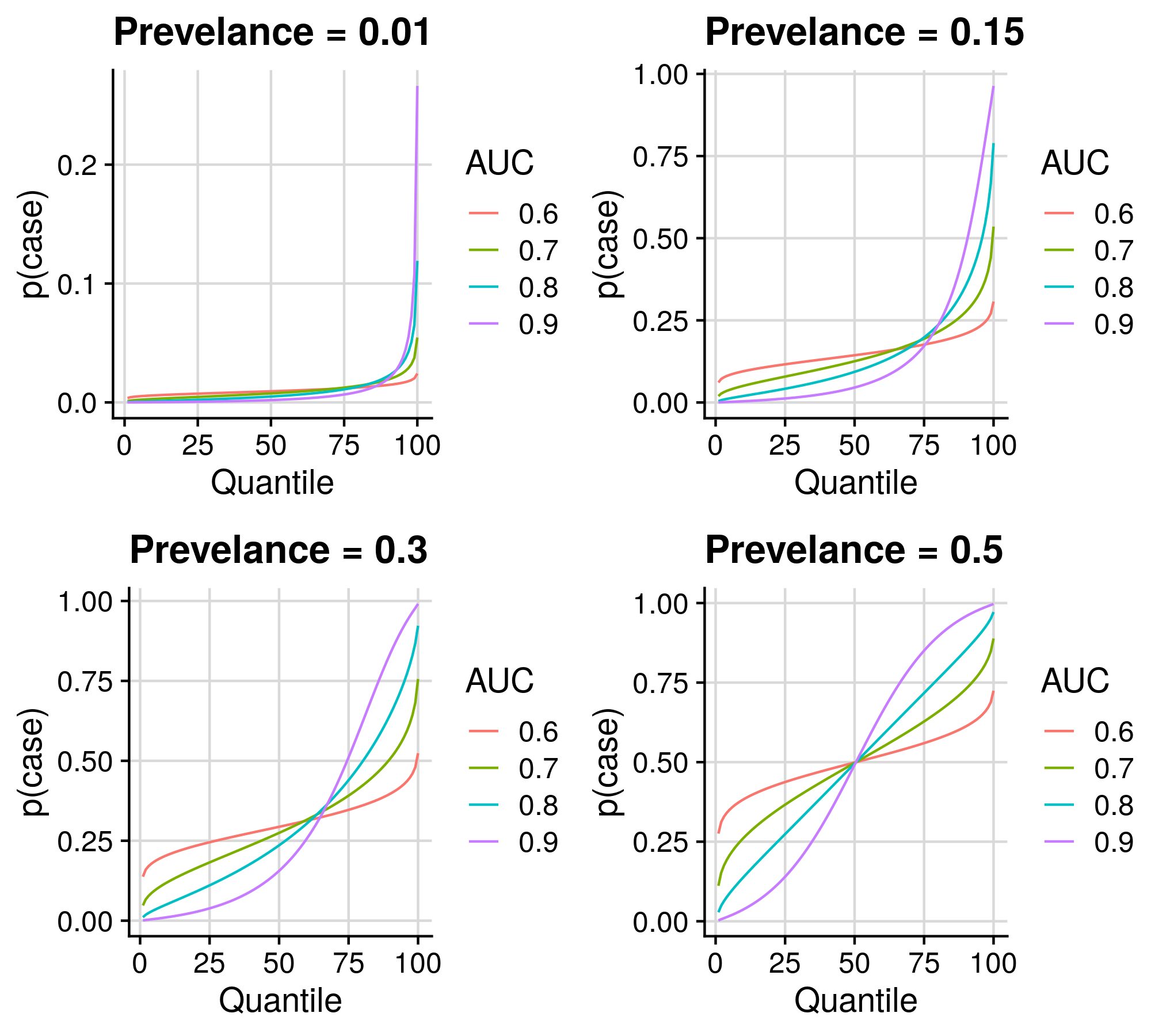


Figure S4. Absolute risk across polygenic score quantiles given a range of polygenic scores AUC and prevalence values. Y-axis shows the proportion of cases per polygenic scores quantile.


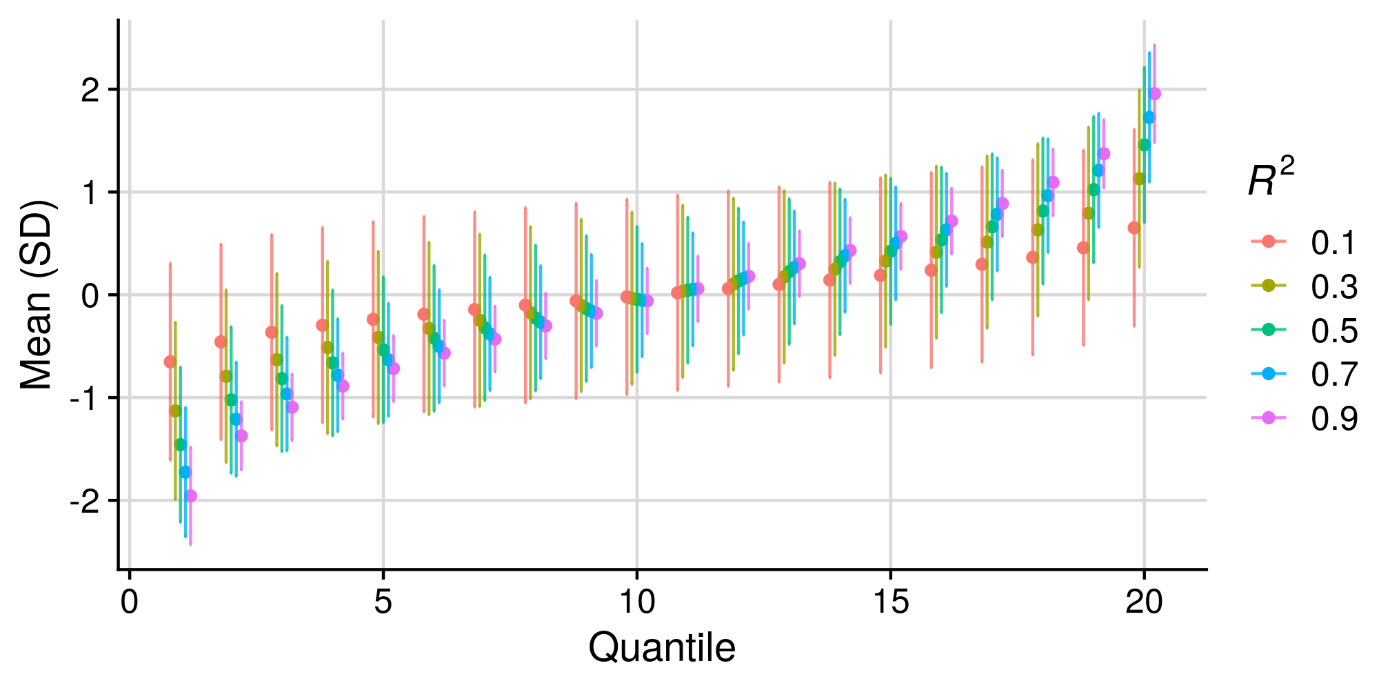


Figure S5. The phenotypic mean and SD across polygenic scores quantiles given a range of polygenic scores R^2^ values.


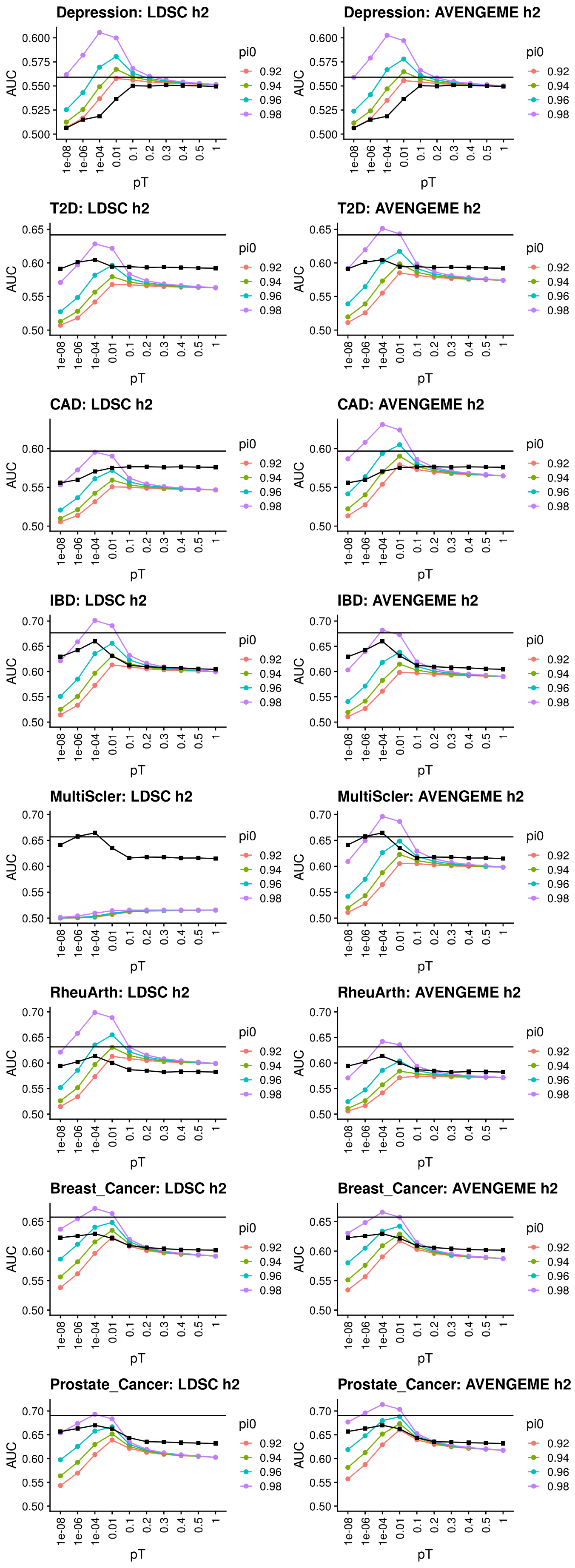


Figure S6. Part 1: Comparison of observed polygenic score AUC values and AVENGEME estimates across p-value thresholds (pT) and observed. The black points indicate the observed AUC when using polygenic scores derived using the pT+clump approach. The horizontal black line indicates the observed AUC of the polygenic score derived using the DBSLMM approach. AVENGEME estimates are provided using a range of pi0 values, indicating the proportion of variants with zero effect. On the left are AVENGEME AUC estimates when using the LDSC SNP-based heritability estimate. On the right are AVENGEME AUC estimates based on AVENGEME estimated SNP-based heritability based on observed pT+clump associations in UK Biobank.


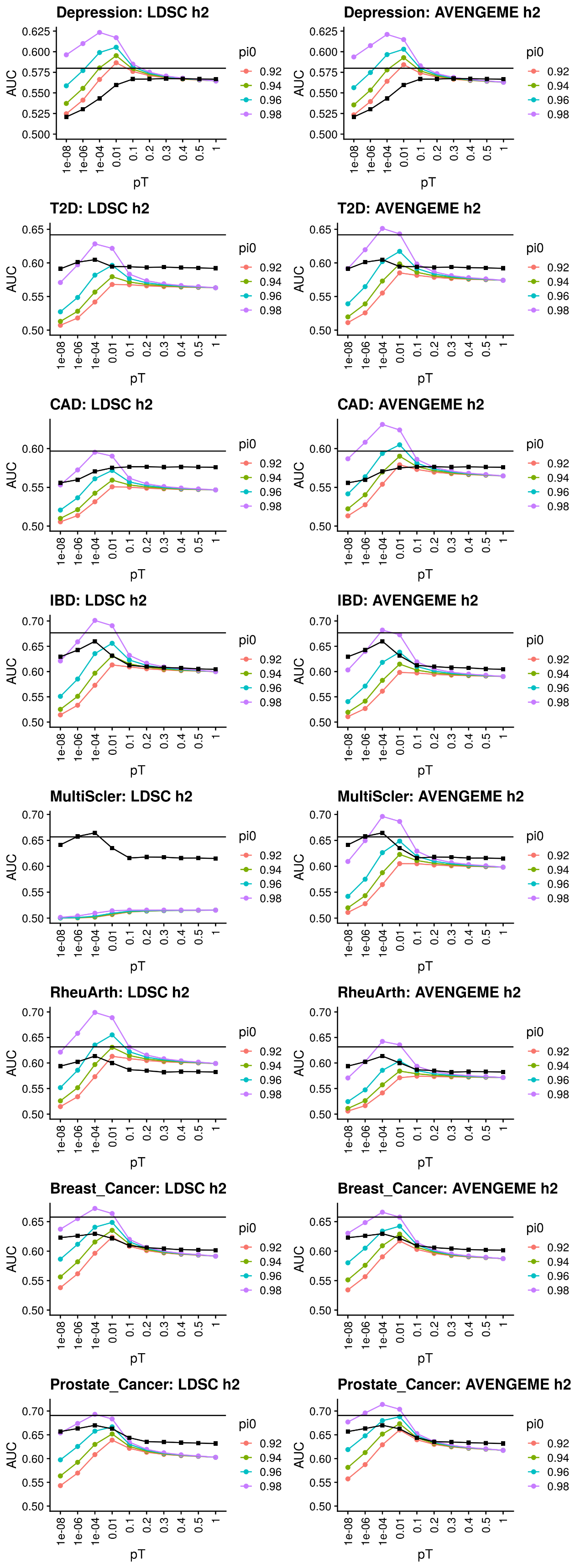


Figure S6. Part 2: Comparison of observed polygenic score AUC values and AVENGEME estimates across p-value thresholds (pT) and observed. The black points indicate the observed AUC when using polygenic scores derived using the pT+clump approach. The horizontal black line indicates the observed AUC of the polygenic score derived using the DBSLMM approach. AVENGEME estimates are provided using a range of pi0 values, indicating the proportion of variants with zero effect. On the left are AVENGEME AUC estimates when using the LDSC SNP-based heritability estimate. On the right are AVENGEME AUC estimates based on AVENGEME estimated SNP-based heritability based on observed pT+clump associations in UK Biobank.


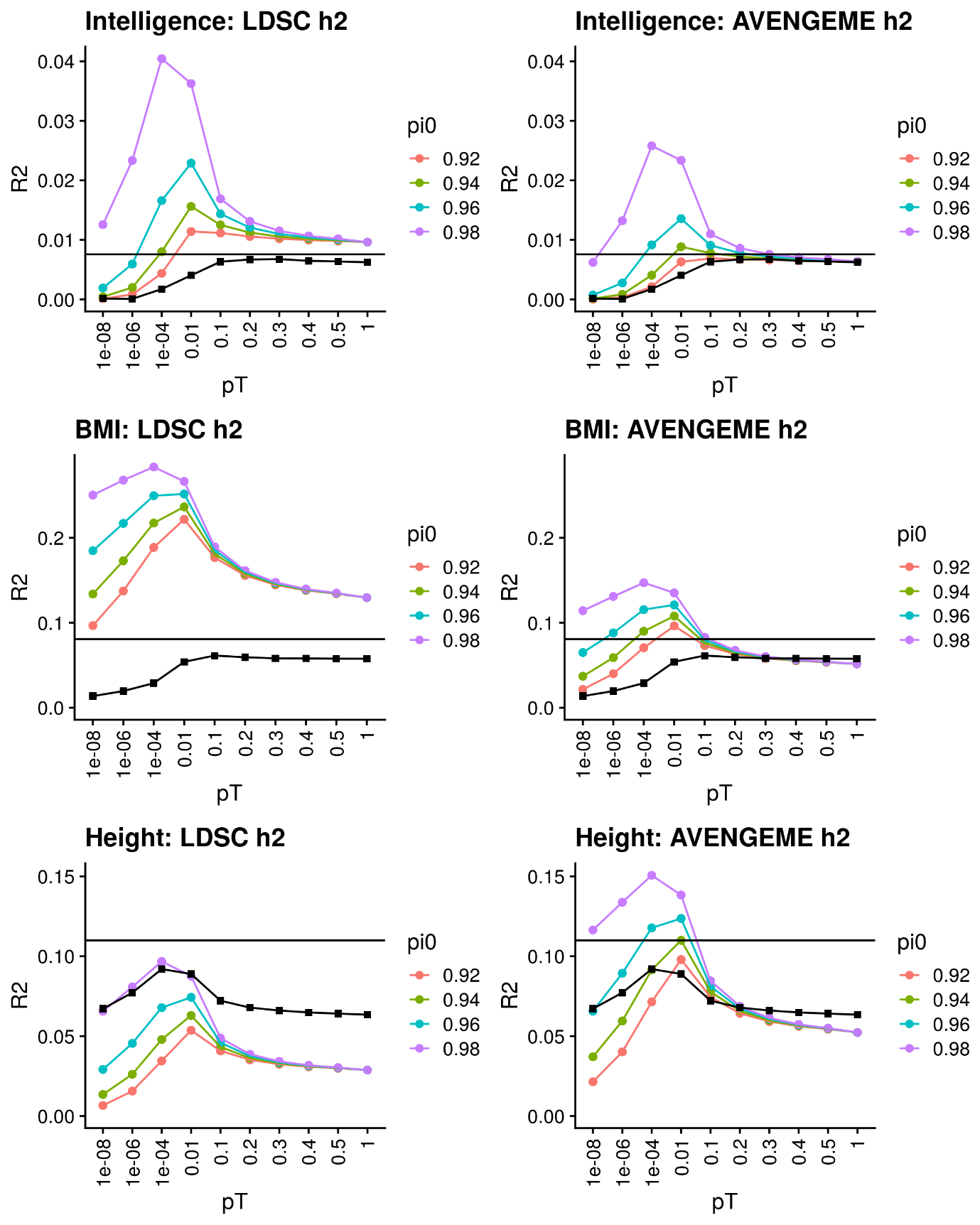


Figure S7. Comparison of observed polygenic score R^2^ values and AVENGEME estimates across p-value thresholds (pT) and observed. The black points indicate the observed R^2^ when using polygenic scores derived using the pT+clump approach. The horizontal black line indicates the observed R^2^ of the polygenic score derived using the DBSLMM approach. AVENGEME estimates are provided using a range of pi0 values, indicating the proportion of variants with zero effect. On the left are AVENGEME R^2^ estimates when using the LDSC SNP-based heritability estimate. On the right are AVENGEME R^2^ estimates based on AVENGEME estimated SNP-based heritability based on observed pT+clump associations in UK Biobank.

## URLs

- LDSC munge_sumstats.py: https://github.com/bulik/ldsc/blob/master/munge_sumstats.py

## References

1. Davis KAS, Coleman JRI, Adams M, Allen N, Breen G, Cullen B, et al. Mental health in UK Biobank–development, implementation and results from an online questionnaire completed by 157 366 participants: a reanalysis. BJPsych open. 2020;6(2):83–90.

2. Smith DJ, Nicholl BI, Breda Cullen DM, Ul-Haq Z, Evans J, Gill JMR, et al. Prevalence and characteristics of probable major depression and bipolar disorder within UK biobank: cross-sectional study of 172,751 participants. PLoS One. 2013;8(11).

3. Glanville KP, Coleman JRI, Hanscombe KB, Euesden J, Choi SW, Purves KL, et al. Classical human leukocyte antigen alleles and C4 haplotypes are not significantly associated with depression. Biol Psychiatry. 2020;87(5):419–30.

4. Fürtjes AE, Coleman JRI, Tyrrell J, Lewis CM, Hagenaars SP. Phenotypic Associations and Shared Genetic Etiology between Bipolar Disorder and Cardiometabolic Traits. medRxiv. 2020;

5. Sudlow C, Gallacher J, Allen N, Beral V, Burton P, Danesh J, et al. UK biobank: an open access resource for identifying the causes of a wide range of complex diseases of middle and old age. PLoS Med. 2015;12(3).

6. Rice ME, Harris GT. Comparing effect sizes in follow-up studies: ROC Area, Cohen’s d, and r. Law Hum Behav. 2005;29(5):615.

7. Wilhelm S, Manjunath GB. tmvtnorm: Truncated Multivariate Normal and Student t Distribution. 2015.

8. Palla L, Dudbridge F. A fast method that uses polygenic scores to estimate the variance explained by genome-wide marker panels and the proportion of variants affecting a trait. Am J Hum Genet. 2015;97(2):250–9.

9. Bulik-Sullivan BK, Loh P-R, Finucane HK, Ripke S, Yang J, Patterson N, et al. LD Score regression distinguishes confounding from polygenicity in genome-wide association studies. Nat Genet. 2015;47(3):291–5.

10. Consortium*† IMSG, ANZgene, IIBDGC, WTCCC2. Multiple sclerosis genomic map implicates peripheral immune cells and microglia in susceptibility. Science (80- ). 2019;365(6460):eaav7188.

11. Patron J, Serra-Cayuela A, Han B, Li C, Wishart DS. Assessing the performance of genome-wide association studies for predicting disease risk. PLoS One. 2019;14(12):e0220215.

12. Lloyd-Jones LR, Zeng J, Sidorenko J, Yengo L, Moser G, Kemper KE, et al. Improved polygenic prediction by Bayesian multiple regression on summary statistics. Nat Commun. 2019;10(1):1–11.
