# Supplementary material for: A tool for translating polygenic scores onto the absolute scale using summary statistics
Source: Eur J Hum Genet. 2022 Jan 4;30(3):339–48. doi: 10.1038/s41431-021-01028-z (PMC8904577; doi:10.1038/s41431-021-01028-z)
Supplement: Supplementary file 2 — Supplementary Table 1 [file 41431_2021_1028_MOESM2_ESM.pdf]

Descriptive statistics for GWAS summary statistics used to predict target sample phenotypes

UKB Phenotype	Code	GWAS Phenotype	Year	PMID	Ncase	Ncontrol	N	h2-obs (SE)	h2-liab (SE)	Intercept
Major Depression	DEPR07	Major depressive disorder (excl. UKB and 23andMe)	2018	29700475	45591	97674	143265	NA	0.1 (0.007)	0.997 (0.009)
Intelligence	COLL01	College completion	2013	23722424	NA	NA	95427	0.105 (0.008)	NA	1.022 (0.009)
BMI	BODY04	BMI	2015	25673413	NA	NA	252064	0.312 (0.006)	NA	0.672 (0.008)
Height	HEIG03	Height	2014	25282103	NA	NA	233681	0.13 (0.014)	NA	1.328 (0.02)
T2D	DIAB05	Type-2 diabetes	2017	28566273	26676	132532	159208	NA	0.118 (0.008)	0.999 (0.009)
CAD	COAD01	Coronary artery disease	2015	26343387	60801	123504	184305	NA	0.055 (0.004)	0.887 (0.008)
IBD	INFB01	Inflammatory Bowel Disease	2015	26192919	12882	21770	34652	NA	0.183 (0.019)	1.07 (0.011)
MultiScler	SCLE03	Multiple sclerosis	2011	21833088	9772	17376	27148	NA	0.02 (0.011)	1.062 (0.009)
RheuArth	RHEU02	Rheumatoid arthritis	2014	24390342	14361	43923	58284	NA	0.143 (0.013)	1.07 (0.008)
Breast Cancer	BRCA01	Breast cancer	2017	29059683	122977	105974	228951	NA	0.15 (0.012)	1.103 (0.013)
Prostate Cancer	PRCA01	Prostate cancer	2017	29892016	79148	61106	140254	NA	0.188 (0.027)	1.081 (0.015)

Note. PMID, PubMed ID; Ncase, Number of cases; Ncontrol, Number of controls; N, Total sample size; h2-obs, SNP-heritability on the observed scale; h2-liab, SNP-heritability on the liability scale; Intercept, LDSC intercept.
