# Supplementary material for: A tool for translating polygenic scores onto the absolute scale using summary statistics
Source: Eur J Hum Genet. 2022 Jan 4;30(3):339–48. doi: 10.1038/s41431-021-01028-z (PMC8904577; doi:10.1038/s41431-021-01028-z)
Supplement: Supplementary file 3 — Supplementary Table 2 [file 41431_2021_1028_MOESM3_ESM.pdf]

Estimated polygenic score AUC using LDSC/AVENGEME approach.

Phenotype	N	AVENGEME h^2	LDSC h^2	LDSC intercept	Prevalence	Sampling	Est. AUC (LDSC h^2)	Est. AUC (AVENGEME h^2)	Obs. AUC (pT1)	Obs. AUC (DBSLMM)
Depression	143265	0.097	0.1	0.997	0.15	0.318	0.567	0.565	0.549	0.559
T2D	152599	0.14	0.118	0.999	0.05	0.168	0.58	0.599	0.592	0.642
CAD	184305	0.078	0.055	0.887	0.03	0.33	0.559	0.59	0.576	0.597
IBD	34652	0.165	0.183	1.069	0.013	0.592	0.631	0.615	0.604	0.677
MultiScler	27148	0.133	0.02	1.062	0.002	0.36	0.515	0.623	0.615	0.657
RheuArth	58284	0.101	0.143	1.065	0.005	0.246	0.631	0.584	0.582	0.632
Breast_Cancer	228951	0.142	0.15	1.103	0.125	0.537	0.635	0.629	0.602	0.658
Prostate_Cancer	140254	0.218	0.188	1.081	0.125	0.564	0.652	0.673	0.632	0.691

Note. N, GWAS sample size; Est. AUC (LDSC h^2), AUC estimated by AVENGEME given SNP-based heritability estimated by LDSC; Prevelance, population prevelance of the outcome; Sampling, sampling fraction of cases and controls in GWAS sample calculated as $N_{case}/(N_{case}+N_{control})$; Est. AUC (AVENGEME h^2), AUC estimated by AVENGEME given SNP-based heritability estimated by AVENGEME; Obs. AUC (pT1), Observed AUC of polygenic scores based on pT+clump polygenic score using a p-value threshold of 1; Obs. AUC (DBSLMM), Observed AUC of polygenic scores based on DBLMM polygenic score.
