# Supplementary material for: A tool for translating polygenic scores onto the absolute scale using summary statistics
Source: Eur J Hum Genet. 2022 Jan 4;30(3):339–48. doi: 10.1038/s41431-021-01028-z (PMC8904577; doi:10.1038/s41431-021-01028-z)
Supplement: Supplementary file 4 — Supplementary Table 3 [file 41431_2021_1028_MOESM4_ESM.pdf]

Estimated polygenic score R^2 using LDSC/AVENGEME approach.

Phenotype	N	AVENGEME h^2	LDSC h^2	LDSC intercept	Obs. R^2 (pT1)	Obs. R^2 (DBSLMM)	Est. R^2 (LDSC h^2)	Est. R^2 (AVENGEME h^2)
Intelligence	95427	0.085	0.105	1.022	0.016	0.009	0.006	0.008
BMI	252064	0.178	0.312	1.328	0.236	0.108	0.058	0.081
Height	233681	0.183	0.13	0.672	0.063	0.11	0.063	0.11

Note. Est. R2 (LDSC h2), AUC estimated by AVENGEME given SNP-based heritability estimated by LDSC; Est. R2 (AVENGEME h2), R2 estimated by AVENGEME given SNP-based heritability estimated by AVENGEME; Obs. R2 (pT1), Observed R2 of polygenic scores based on pT+clump polygenic score using a p-value threshold of 1; Obs. R2 (DBSLMM), Observed R2 of polygenic scores based on DBLMM polygenic score.
